# An Optimized and Sensitive Pharmacokinetic Quantitative Method of Investigating Gastrodin, Parishin, and Parishin B, C and E in Beagle Dog Plasma using LC-MS/MS after Intragastric Administration of Tall *Gastrodia* Capsules

**DOI:** 10.3390/molecules22111938

**Published:** 2017-11-10

**Authors:** Junqiu Liu, Sha Chen, Jintang Cheng, Jun Zhang, Yuesheng Wang, An Liu

**Affiliations:** Key laboratory of Beijing for identification and safety evaluation of Chinese medicine, Institute of Chinese Materia Medica, China Academy of Chinese Medical Sciences, No. 16, Nanxiaojie, Dongzhimennei, Beijing 100700, China; liujoal@163.com (J.L.); schen@icmm.ac.cn (S.C.); jtcheng@icmm.ac.cn (J.C.); jzhang@icmm.ac.cn (J.Z.); yswang@icmm.ac.cn (Y.W.)

**Keywords:** gastrodin, parishin, tall *Gastrodia* capsules, pharmacokinetics, correlation, LC-MS/MS

## Abstract

*Gastrodia elata* Blume, called Tianma in China, has been widely used to treat headaches, convulsions and epilepsy for thousands of years. In the present study, a series of optimizations were employed to develop a rapid, sensitive, and reliable high-performance liquid chromatography-triple quadrupole mass spectrometry method, which was then used for the simultaneous determination of gastrodin, parishin, parishin B, parishin C and parishin E in beagle dog plasma after intragastric administration of tall *Gastrodia* capsules (Tianma brand). The chromatographic separation was achieved on a C_18_ column with gradient elution by using a mixture of 0.4% formic acid aqueous solution and acetonitrile as the mobile phase at a flow rate of 0.15 mL/min. A tandem mass spectrometric detection was conducted using multiple-reaction monitoring (MRM) via electrospray ionization (ESI) source in negative ionization mode. Samples were pre-treated by a single-step protein precipitation with methanol, and bergenin was used as internal standard (IS). Under the optimized conditions, the lower limit of quantification (LLOQ) was 0.10 ng/mL for gastrodin, 0.40 ng/mL for parishin B, 0.02 ng/mL for parishin E and 0.20 ng/mL for parishin and parishin C, all of which previously were the highest levels of sensitivity. The methods were optimized for selectivity, calibration curves, accuracy and precision. Extraction recoveries, matrix effects and stability were within acceptable ranges. Pharmacokinetic parameters of the tested substances were also quantitatively determined. Finally, a possible metabolic pathway was induced based on correlations obtained from quantitative and qualitative data analysis in vivo.

## 1. Introduction

Pharmacokinetic studies on *Gastrodia elata* Blume (GE, tianma) have mainly focused on gastrodin and one or two parishin and hydroxybenzyl alcohol compounds in dogs and rats [1,2,3]. To date, pharmacokinetic research on compounds like gastrodin, *p*-hydroxybenzyl alcohol and parishin compounds has typically been conducted in rodents. Some bottlenecks exist however in studying the oral administration pharmacokinetics of these compounds in plasma, such as a lack of a highly sensitive method of integrated analysis of gastrodin and parishins, or a clear method for explaining the inner links among these bioactive compounds in vivo. In addition to the high quantitative limit and transformation of compounds, suitable acidity of the mobile phase is also key for bio-availability analysis in vivo for GE compounds analysis. However, the mobile phase acidity can change the charged condition of ions, also influencing the sensitivity of quantitative methods. Thus, there is an urgent need to establish a systematic analysis protocol.

Research on GE has recently focused primarily on metabolism. It was shown that in vivo, parishin can be biotransformed into a variety of other metabolites including parishin B, parishin E, and parishin G [4]. While gastrodin and parishin have been the subject of research, there have however been no pharmacokinetic studies of parishin and its analogues. Both gastrodin and parishin are potentially active compounds which may have therapeutic effects in vivo. To gain a better understanding of the molecular mechanisms of GE, it is therefore necessary to investigate the pharmacokinetics of many of its components.

Tall *Gastrodia* capsules are a traditional Chinese medicine composed of the rhizomes of the GE plants. GE is a prominent and effective traditional Chinese herb that has been used as a neuroprotectant [5], anticonvulsant [6], anti-asthma drug [7], anti-inflammatory and analgesic [8,9,10,11]. More than sixty compounds been characterized have to date in GE, including some characteristic parishin compounds [12,13]. Previous studies have shown that gastrodin is the most active component in GE [14,15,16,17,18,19]. Parishin as well as parishin B, C, E, and F and some other structural analogs have been identified or isolated in the GE rhizome as potential active components in vivo. Further research is however required to establish the relationship between the main active gastrodin and parishin compounds, and to identify any transformation of the main compounds which may occur in vivo. In the present study, a systematic and reliable pharmacokinetic quantitative method was developed and validated, and a correlation analysis was conducted between the bioactive compounds in beagle dog plasma.

## 2. Results and Discussion 

### 2.1. Sample Preparation

SP 2 was used as a solvent to analyze parishin compounds in GE, especially with regards to pure parishin. SP 3 and SP 4 were based on the results from SP 1 and SP 2, which showed that the target compounds were obtained with good separation efficiency. Formic acid (1%) was added into SP 5–8, considering the improvement of all the detecting compounds. SP 5, methanol with 1% formic acid was ultimately chosen as the best-performing extraction solvent due to good analyte separation and high extraction recovery (>90%). In the plasma sample extraction recovery was higher than 90% when using the five-fold volume of extraction solvent. Under these conditions, the interference with endogenous compounds was significantly inhibited.

### 2.2. Elution System

**Figure 1:**
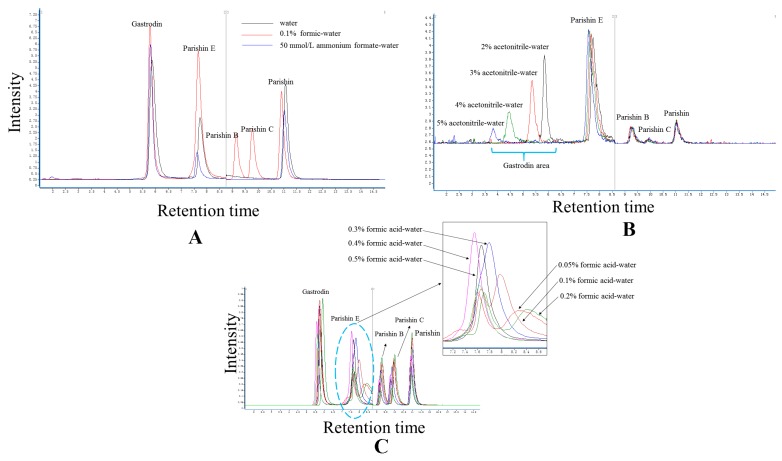
Optimization of elution system. Comparison was presented on the base of total ions mass spectrograms. (**A**) Type of aqueous phase; (**B**) Percentage of initial mobile phase; (**C**) Acidity in aqueous phase.

Parishin B and C were well detected in the aqueous phase of the 0.1% formic acid-water combination, but not in the other two aqueous phases (Figure 1A). The peak area of parishin E increased significantly in this condition. Because of this, 0.1% formic acid-water was chosen as the aqueous phase for further analyses.

The different percentages of the initial mobile phase were important to the matrix effects in some of the target compounds and also played a substantial role in improving the sensitivity, making optimization of the initial mobile phase crucial. As the present study used gastrodin as the water-soluble compound due to its ready inhibition by the endogenous protein, the initial mobile phase was the key condition needing to be optimized. Accordingly, the initial mobile phase was preferable in the following course of optimization. In our study, there was a positive correlation between the sensitivity of gastrodin and the percentage of aqueous phase in the initial mobile phase. Finally, 2% acetonitrile-water was chosen as the optimal initial mobile phase condition (Figure 1B). However, the high acidity of this phase indicated that acidity may be an important factor affecting the resolution, which may in turn affect recovery. In the present study, all candidates were tested with different acid percentages. The findings showed that 0.4% formic acid was superior to the other candidates (Figure 1C). The data (peak area) were presented in Supplementary materials (Table S1).

### 2.3. Obtaining Compounds in Plasma Using LC-MS/MS

Mass ion source parameters were also optimized to maximize sensitivity. All measurements were performed in negative ion mode. This experiment was carried out using automatic injection of the mixture that contained the test compounds in 10 μL volumes. The optimization of drying gas temperature and flow rates, sheath gas temperature and flow rates, and nozzle voltage were chosen empirically (Table 1 and Figure S1).

### 2.4. Method Validation

#### 2.4.1. Separation, Selectivity, and Sensitivity

After conducting numerous pre-experiments to establish optimization of bio-sample pretreatments, a well optimized LC-MS/MS method was established that resulted in good peak shape with baseline separation for all analytes with baseline separation from the endogenous plasma components. The method obtained high selectivity with the mass transitions of gastrodin, parishin, parishin B, parishin C, parishin E and bergenin. All analytes were monitored in negative mode.

#### 2.4.2. Recovery and Matrix Effect

The extraction efficiency for gastrodin, parishin, and parishin B, C and E in beagle dog plasma was investigated by measuring the recovery. According to the sample concentration, three concentrations (high, medium and low) of standard solutions were added into known amounts of the sample solution. These were then extracted and subjected to quantitative analysis as described above. Each standard was tested in triplicate at each concentration. Extraction recovery of all analytes ranged from 95.3% to 101%, indicating that the extraction method had close to optimal accuracy. In addition, the relative standard deviation (RSD) values for all analytes (<6.93%) indicated good reproducibility. Matrix effects of all analytes ranged from 96.6% to 107% (*n* = 6). The IS value at a concentration level of 0.09408 μg/mL was 102 ± 2.36 (*n* = 6). The results showed that the matrix effect on the analysis of the five analytes and the IS in beagle dog plasma was negligible. Results are shown in Table 2.

#### 2.4.3. Precision and Accuracy

Six samples were extracted and analyzed on three consecutive days to determine the inter-day precision, while six samples were extracted and evaluated on the same day to determine the intra-day precision. Intra- and inter-day precision were assessed using the RSD values of all compounds. All values were below 4.50%, and accuracy deviation was within 98.2 ± 6.0% of the actual values at each QC level. These results suggest that the accuracy and precision in the present assay were acceptable for analysis.

#### 2.4.4. Stability

Stability under different storage conditions was assessed for all analytes. Analytes in beagle dog plasma were stable at −20 °C for 30 days for three freeze-thaw cycles (%RE, ±5%) and at room temperature (%RE, ±15%). For reconstituted solutions, the stability of all analytes showed no significant degradation when stored in the autosampler for 48 h (%RE, ±6%).

### 2.5. Linearity of Calibration Curves and LLOQs

Linear regression modeling of the ratio of analytes to the internal standard (*y*) and plasma concentration (*x*) for gastrodin yielded the equation *y* = 3.1278*x* + 139.87 (1.32–4800 ng/mL, 0.9990); for parishin, *y* = 1.6481*x* − 18.496 (18–1800 ng/mL, 0.9987); for parishin B, *y* = 1.3725*x* − 14.674 (18–2000 ng/mL, 0.9998); for parishin C, *y* = 1.8269*x* − 8.9595 (6–500 ng/mL, 0.9987); and for parishin E, *y* = 22.974*x* − 73.557 (4–400 ng/mL, 0.9997). The correlation coefficient of all analytes was found to be >0.990, indicating good linearity. LLODs for the above compounds were 0.35, 0.83, 1.46, 0.97 and 0.067 ng/mL, respectively. These were obtained under conditions where precision and accuracy were below 20% and the signal-to-noise ratio was 10. 

### 2.6. Application to Pharmacokinetic Studies

**Figure 2:**
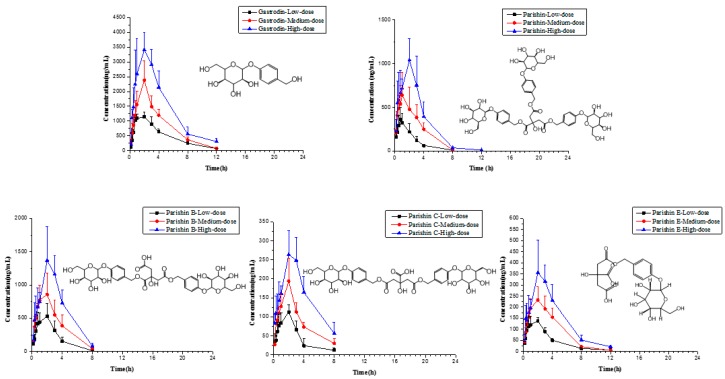
Mean plasma concentration-time profile of five compounds in beagle dog after intragastric administration of low dose, medium dose and high dose tall *Gastrodia* capsules (*n* = 5).

After full validation, the LC-ESI-MS/MS method was used to analyze large batches of beagle dog plasma. In the pharmacokinetic study, we successfully and effectively investigated gastrodin, parishin and parishin B, C and E from intragastrically administered tall *Gastrodia* capsules at low (0.125 g/kg), medium (0.25 g/kg) and high doses (0.5 g/kg). The corresponding pharmacokinetic parameters were generated by fitting plasma concentration profiles to a non-compartmental model. These are summarized in Table 3, expressed as the mean ± SD (*n* = 5). The mean plasma concentration-time curves (*n* = 5) of five analytes are shown in Figure 2.

Gastrodin was detected in beagle dog plasma 10 min after intragastric administration of capsules at all three dosage levels. For low, medium and high doses, retention time of the maximum plasma concentration (T_max_) was reached at 1.10, 1.75 and 2.00 h, respectively. These results were similar to the findings of earlier studies [1,20]. However, T_max_ in the present study was roughly double that found in rats [21]. This shows that the onset time of effects from gastrodin in beagle dogs is longer than that found in rats. In addition, T_max_ for the high dose condition was twice long as in the low dose condition, indicating that the amount of gastrodin in vivo may influence the time of maximum plasma concentration. The area under plasma concentration-time curve from 0 to infinity (AUC_0–t_) of gastrodin was 4890 ± 2060, 8830 ± 1860 and (17.0 ± 4.24) × 10^3^ ng/mL·h for low, medium and high doses, respectively, and area under plasma concentration-time curve from 0 to infinity (AUC_0–∞_) was 5080 ± 2110, 9170 ± 1790 and (17.6 ± 4.71) × 10^3^ ng/mL·h. Comprehensive area under plasma concentration-time curve (AUC) analysis of all five analytes showed that gastrodin was the constituent with the highest concentration in plasma. The concentration of parishin B was one-third the concentration of gastrodin, and the other three compounds had lower concentrations.

Several previous studies of GE have focused primarily on gastrodin [22,23,24]. However, both parishin and parishin B are also potentially active compounds based on their high concentrations in vivo and should therefore be investigated more deeply at a pharmacological level. At the same time Jiang et al. reported [4] that gastrodin can be detected in heart, liver and other tissues, which could be expanded the pharmacodynamics of gastrodin study. Mean plasma clearance (CL) for gastrodin, parishin, and parishin B, C and E was 0.2, 3.14, 0.87, 0.96 and 1.61 L/h/kg, respectively, showing that gastrodin was eliminated more rapidly than any of the other compounds in beagle dogs. The maximum plasma concentration (C_max_) of gastrodin was 1200 ± 120, 2050 ± 495 and 3760 ± 778 ng/mL in the low, medium, and high intragastric administration levels, respectively. Besides, for the three dosage levels, mean residence time from 0 to 24 h (MRT_0–t_) was 2.98 ±1.01, 3.46 ± 0.29 and 3.54 ± 0.69 h; mean residence time from 0 to infinity (MRT_0–∞_) was 3.33 ±1.09, 3.80 ± 0.28 and 3.93 ± 0.99 h; and terminal elimination half-life (t_1/2_) was 1.86 ± 0.67, 1.90 ± 0.26 and 2.09 ± 0.68 h. 

### 2.7. Correlation Analysis

**Figure 3:**
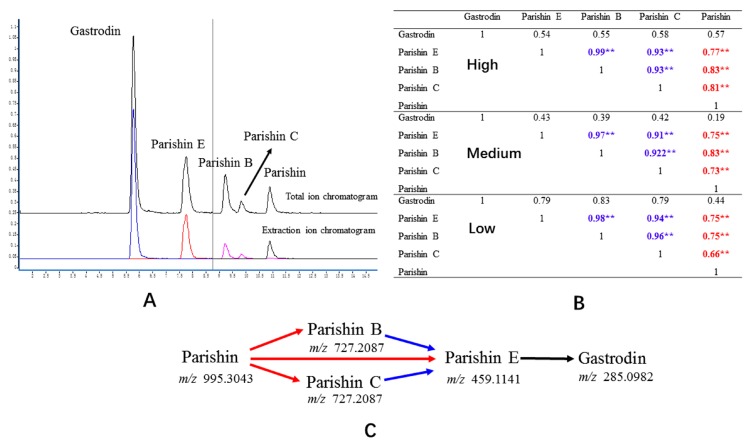
(**A**) The total ion mass spectrograms and extraction ion mass spectrogram of the potential compounds in beagle plasma after administration of tall *Gastrodia* capsules; (**B**) Correlation coefficients of five compounds (** represents *p* < 0.01); (**C**) Putative pathway from parishin to gastrodin in vivo.

Resultant pharmacokinetic data were then used to assess correlations between the four parishin compounds and gastrodin in vivo. Correlation analysis was conducted using SPSS 16.0 software. Pearson’s correlation coefficient was used to investigate correlations among the five compounds. Correlation coefficients were low among the five compounds at all doses (mean: 0.54). However, stronger correlations were found between parishin, parishin B, parishin C and parishin E. Parishin and parishin B, parishin and parishin C, and parishin and parishin E comprised Group 1 (mean: 0.76). Parishin B and parishin E, and parishin C and parishin E comprised Group 2 (mean: 0.95). These correlation coefficients (Figure 3) suggest that gastrodin and parishin compounds can be biotransformed into each other at different rates in vivo. This finding is consistent with previous reports of biotransformation between parishins and gastrodin [4], furthermore, our detected compounds were also reported by Tang et al. [25] using the Q-TOF-MS/MS.

## 3. Experimental

### 3.1. Materials and Reagents

Reference compounds of parishin and parishin B, C and E were purchased from Jiangxi Herbfine Hi-Tech (Nanchang, Jiangxi, China). Bergenin as internal standard (IS) and gastrodin were purchased from the National Institute for the Control of Biological and Pharmaceutical Drugs (Beijing, China). The purity of reference substances was above 98%. Tall *Gastrodia* capsules were kindly supplied by Guizhou Eakan Pharmaceutical (Guiyang, China). Methanol and acetonitrile of MS grade were acquired from Sigma-Aldrich (St. Louis, MO, USA) and formic acid used for HPLC-MS analysis from Anaqua Chemicals Supply (Houston, TX, USA). Ultrapure water was produced using a Milli-Q water purification system (Millipore, Bedford, MA, USA). All other reagents used in the present study were analytical grade.

### 3.2. Instruments and Analytical Conditions

Quantitative analysis was conducted on a 1290 UPLC-photodiode array 6460 triple quadrupole mass spectrometry system (LC-MS/MS, Agilent Technologies, Palo Alto, CA, USA). Analysis was carried out on. Separation of analytes was performed on a reversed phase Agilent Zorbax XDB-C_18_ HPLC column (2.1 mm × 50 mm, particle size 3.5 μm) with the column oven set to 30 °C. The elution gradient was carried out with a binary solvent system consisting of an aqueous solution of 0.4% *v*/*v* formic acid in water (A) and acetonitrile (B) at a flow rate of 0.15 mL/min. Optimized separation was obtained according to a linear gradient (0–2 min, 2% B; 2–4 min, 2–12% B; 4–8 min, 12–20% B; 8–14 min, 20–28% B; 12–15 min, 28–90% B). MS/MS conditions were as follows: negative ion mode, drying gas (N_2_) flow rate 8.0 L/min, drying gas temperature 300 °C, declustering potential 150 V, nebulizer pressure 45 psi, ESI Vcap 3500 V, sheath gas temperature 300 °C, sheath gas flow 12 L/min, nozzle voltage 500 V. The mass transition of gastrodin (*m*/*z* 331.1→123.0, CE: 3 eV), parishin (*m*/*z* 995.4→727.0, CE: 42 eV), parishin B (*m*/*z* 727.3→423.0, CE: 20 eV), parishin C (*m*/*z* 727.3→423.1, CE: 20 eV), parishin E (*m*/*z* 459.3→110.9, CE: 19 eV) and bergenin (*m*/*z* 327.0→234.0, CE: 4 eV).

### 3.3. Animal Experiments

Adult male Sprague-Dawley (SD) beagle dogs (12 kg ± 0.020 kg) were purchased from Beijing Shahe Bio-Technology (Beijing, China; certificate No. SCXK2014-0007). Before drug administration, the dogs were fasted for 12 h with free access to water. They were maintained on a 12 h light-dark cycle (light on from 6:00 to 18:00 h) at ambient temperature (22–24 °C) with 60% relative humidity. The animals were observed twice daily and body weights were recorded once per day to assess their general health. All experimental protocols on animals were carried out according to the Guidelines of the Committee on the Care and Use of Laboratory Animals of China.

### 3.4. Drug Administration and Blood Sampling

Fifteen beagle dogs were randomly divided into three groups to receive an intragastric dose of tall *Gastrodia* capsules (0.125, 0.25 and 0.5 g/kg). Capsules were ultrasonically dissolved in pure water and suspended at concentrations of 5, 10 and 20 mg/mL for intragastric administration. The concentration of gastrodin, parishin, parishin B, parishin C are measured at 3.29 mg/g, 4.482 mg/g, 2.258 mg/g, 0.69 mg/g, respectively. The HPLC quantitative analysis was based on the previous report with some modification [26]. After administration, serial blood samples (about 2.00 mL) were collected from the foreleg vein (the ophthalmic artery plexus) into heparin-containing capillary tubes at 10, 20, 30, 45, 60, 120, 180, 240, 480, 720 and 1440 min. Blood samples were promptly centrifuged at 5000 rpm for 5 min, and the plasma was separated and stored at −20 °C until analysis.

### 3.5. Experimental Condition Optimization

The aims of the optimization experiment for LC-MS/MS were not only to obtain higher analysis efficiency with good recovery, but also to remove matrix interference. The optimization of LC-MS/MS conditions for all tested compounds was achieved in vivo after intragastric administration of tall *Gastrodia* capsules. Different candidate systems of sample preparation (SP)/elution system (ES) combinations were used for optimization.

Based on analysis of relevant previous research [1,2,3], eight candidate sample preparation systems with different organic solvents were tested to optimize the five quantitative compounds and bergenin in plasma samples (hereafter SP 1–8). Sample preparation procedures were classified into four groups. For Group 1 (SP 1, SP 5) methanol was used as the extraction solvent; for Group 2 (SP 2, SP 6), acetonitrile; for Group 3 (SP 3, SP 7), a 1:1 mixture of methanol and ethanol; for Group 4 (SP 4, SP 8), a 1:1:1 mixture of methanol, acetonitrile and acetone. In addition, SP 5–8 were combined with 1% formic acid, whereas SP 1–4 were not.

The elution systems included an aqueous phase and an initial mobile phase. The acidity of the mobile phase was also optimized. The three candidate elution systems (ES) consisted of water, 0.1% formic acid-water and 50 mmol/L ammonium formate-water, which were commonly used in previous studies but optimized here.

The acidity of the mobile phase was also an important factor for the analysis of gastrodin, parishin, and parishin B, C and E. Different levels of acidity were achieved through the addition of 0.05, 0.1, 0.2, 0.3, 0.4, or 0.5% formic acid into the water phase, and were compared when assessing the results.

### 3.6. Plasma Sample Preparation

Plasma samples were thawed to 4 °C before processing. An aliquot of 200 μL of the plasma sample was transferred into an Eppendorf tube, and 1000 μL of ice-cold 1% formic acid methanol containing IS (10 μL, 2.0 μg/mL) was added to precipitate the plasma proteins. Samples were then vortexed for 3 min and centrifuged at 12,000 rpm for 10 min. The supernatant was transferred and evaporated to dryness under a gentle stream of nitrogen at 40 °C. The residue was reconstituted in 200 μL of 5.0% methanol–water, and a 10 μL aliquot was injected for analysis.

### 3.7. Validation of the Method

Specificity and selectivity were evaluated by analyzing chromatogram comparisons between blank plasma, blank plasma spiked with IS/analytes, and dog plasma samples. Calibration curves were established from peak area ratios (analyte/IS) versus concentrations. QC samples at low, medium and high concentrations were designed to assess the intra- and inter-day precision. In addition, stability (short- and long-term stabilities), extraction recoveries, limit of detection (LOD), limit of quantification (LOQ) and linearity were also investigated. 

### 3.8. Pharmacokinetic Study and Data Analysis

The pharmacokinetic parameters were calculated for each subject using the Drug and Statistics (DAS) software package (version 3.0; www.drugchina.net). Parameters consisted of maximum plasma concentration (C_max_), corresponding time (T_max_), area under plasma concentration-time curve from 0 to 24 h (AUC_0–t_), area under plasma concentration-time curve from 0 to infinity (AUC_0-∞_), mean residence time from 0 to 24 h (MRT_0–t_), mean residence time from 0 to infinity (MRT_0–∞_), terminal elimination half-life (t_1/2_) and plasma clearance (CL). A non-compartmental model was used to calculate the parameters. Results are presented as the mean ± standard deviation (S.D.).

Correlation analysis was conducted using SPSS 16.0 for Windows (IBM, Armonk, NY, USA). Pearson’s correlation coefficient was used to investigate correlations between the five compounds.

## 4. Conclusions

A variety of studies in recent years have investigated different methods to establish the presence of gastrodin in plasma samples. One study found that LLOQs of UV detection reached 100 ng/mL [21]. Research has also validated the use of fluorescence detection for quantification of parishin and its metabolites in rats at LLOQs of 2.5 ng/mL [27]. Some studies investigated the comparative pharmacokinetics of parishin, gastrodin, *Gastrodia elata* extract and Rhizoma Gastrodiae capsules using a LC-MS method [21]. Researchers have investigated the holistic pharmacokinetics of gastrodin injection and Rhizoma Gastrodiae capsules, but did not conduct a detailed analysis. In the present study, a rapid, sensitive and reliable LC-MS/MS method was for the first time developed and validated for the simultaneous determination of five active and potential compounds of tall *Gastrodia* capsules in beagle dog plasma. 

In this present study, a validated LC-MS/MS method was firstly used for simultaneous determination of five bioactive compounds in dog plasma. Correlation analysis method was firstly used to analysis the correlation between gastrodin and parishin compounds. Preliminary experiments allowed optimization to the point was a good separation of two isomers (parishin B and parishin C) was successfully obtained. With this excellent sensitive, accurate and fast bioanalytical method, a good and reliable pharmacokinetic study was established. The results might be helpful for investigating the bioactivity mechanism and clinical application of Tianma.

## Figures and Tables

**Table 1:** Six different ion source parameter combinations.

	Condition 1	Condition 2	Condition 3	Condition 4	Condition 5	Condition 6
Drying gas Temperature (°C)	300	300	300	300	300	300
Drying gas flow rates (L/min)	5	8	8	8	8	8
Sheath gas temperature (°C)	250	400	300	300	350	350
Sheath gas flow rates (L/min)	9	12	12	12	12	12
Nozzle voltage (kV)	0.5	1.0	1.0	0.5	0.5	1.0

**Table 2:** Recovery and matrix effect of gastrodin, parishin, parishin B, parishin C and parishin E in beagle dog plasma (*n* = 6).

Spiked Conc. (ng/mL)		Recovery (%)	RSD (%)	Matrix Effect (%)	RSD (%)
Gastrodin	3620	98.9	0.84	101	2.89
	723	99.5	0.81	107	1.90
	144	98.1	1.46	99.9	4.45
Parishin	840	98.4	2.83	103	1.91
	168	101	2.43	107	3.82
	33.6	97.0	7.65	102	8.02
Parishin B	1040	95.3	3.08	103	2.24
	208	95.3	4.81	96.6	2.00
	41.6	98.5	5.19	103	4.64
Parishin C	200	98.9	2.09	101	4.72
	40	96.5	5.55	100	6.35
	8	97.5	6.93	100	10.8
Parishin E	306	96.3	2.33	99.8	1.87
	61.2	98.5	2.14	101	1.56
	12.2	99.2	3.54	100	2.65

**Table 3:** The mean plasma pharmacokinetic parameters under low, medium and high doses of the five analytes (*n* = 5).

Compounds	Parameter	Unit	Low Dose	Medium Dose	High Dose
Gastrodin	AUC_(0–t)_	ng/mL·h	4890 ± 2060	8830 ± 1860	(17.0 ± 4.24) × 10^3^
	AUC_(0–∞)_	ng/mL·h	5080 ± 2110	9170 ± 1790	(17.6 ± 4.71) × 10^3^
	MRT_(0-t)_	h	2.98 ± 1.01	3.46 ± 0.29	3.54 ± 0.69
	MRT_(0–∞)_	h	3.33 ±1.09	3.80 ± 0.28	3.93 ± 0.99
	t_1/2_	h	1.86 ± 0.67	1.90 ± 0.26	2.09 ± 0.68
	CL	L/h/kg	0.21 ± 0.13	0.20 ± 0.04	0.21 ± 0.05
	T_max_	h	1.10 ± 0.52	1.75 ± 0.90	2.00 ± 0.71
	C_max_	ng/mL	1200 ± 120	2050 ± 495	3760 ± 778
Parishin	AUC_(0–t)_	ng/mL·h	868 ± 314	1500 ± 836	3270 ± 1370
	AUC_(0–∞)_	ng/mL·h	899 ± 299	1520 ± 832	3280± 1370
	MRT_(0–t)_	h	1.80 ± 0.42	2.49 ± 0.40	2.48 ± 0.36
	MRT_(0–∞)_	h	2.00 ± 0.30	2.65 ± 0.44	2.50 ± 0.35
	t_1/2_	h	1.15 ± 0.18	1.38 ± 0.34	1.05 ± 0.16
	CL	L/h/kg	2.69 ± 1.01	3.56 ± 1.75	3.18 ± 1.65
	T_max_	h	1.00 ± 0.56	1.55 ± 0.62	1.45 ± 0.76
	C_max_	ng/mL	388 ± 116	462 ± 230	969 ± 292
Parishin B	AUC_(0–t)_	ng/mL·h	1550 ± 530	2700 ± 1130	4700 ± 2330
	AUC_(0–∞)_	ng/mL·h	1570 ± 533	2720 ± 1140	4720 ± 2330
	MRT_(0–t)_	h	2.25 ± 0.26	2.78 ± 0.42	2.76 ± 0.44
	MRT_(0–∞)_	h	2.32 ± 0.25	2.84 ± 0.44	2.80 ± 0.42
	t_1/2_	h	1.08 ± 0.11	1.18 ± 0.29	1.17 ± 0.22
	CL	L/h/kg	0.72 ± 0.39	0.78 ± 0.23	1.11 ± 0.84
	T_max_	h	1.25 ± 0.69	1.80 ± 0.45	2.00 ± 0.71
	C_max_	ng/mL	526 ± 143	746 ± 320	1220 ± 562
Parishin C	AUC_(0–t)_	ng/mL·h	349 ± 100	567 ± 198	1090 ± 523
	AUC_(0–∞)_	ng/mL·h	359 ± 99.3	578 ± 200	1100 ± 527
	MRT_(0–t)_	h	2.47 ± 0.28	3.14 ± 0.54	3.18 ± 0.69
	MRT_(0–∞)_	h	2.70 ± 0.28	3.31 ± 0.59	3.25 ± 0.72
	t_1/2_	h	1.37 ± 0.18	1.56 ± 0.41	1.29 ± 0.36
	CL	L/h/kg	0.72 ± 0.23	0.91± 0.23	1.26 ± 1.05
	T_max_	h	1.35 ± 0.60	2.00 ± 0.71	2.40 ± 0.55
	C_max_	ng/mL	107 ± 29.4	144 ± 55.1	243 ± 99.6
Parishin E	AUC_(0-t)_	ng/mL·h	545 ± 188	865 ± 257	1620 ± 76
	AUC_(0-∞)_	ng/mL·h	553 ± 190	868 ± 254	1630 ± 759
	MRT_(0-t)_	h	3.86 ± 1.05	3.74 ± 0.66	4.36 ± 0.59
	MRT_(0-∞)_	h	4.19 ± 1.36	3.79 ± 0.60	4.52 ± 0.66
	t_1/2_	h	3.09 ± 2.03	1.72 ± 0.13	3.00 ± 1.71
	CL	L/h/kg	1.33 ± 0.82	1.50 ± 0.35	2.01 ± 1.44
	T_max_	h	1.35 ± 0.60	2.00 ± 0.71	1.80 ± 0.45
	C_max_	ng/mL	136 ± 35.3	192 ± 57.0	312 ± 138

## Supplementary Materials

Supplementary materials are available online. Figure S1: Total ions mass spectrograms obtained from different ion source parameters. Table S1: Intensity of gastrodin, parishin, parishin B, parishin C and parishin E under different experimental condition.

## References

[1] Jia Y., Li X., Xie H., Shen J., Luo J., Wang J., Wang K.D., Liu Q., Kong L. (2014). Analysis and pharmacokinetics studies of gastrodin and *p*-hydroxybenzyl alcohol in dogs using ultra fast liquid chromatography-tandem mass spectrometry method. J. Pharm. Biomed. Anal..

[2] Tang C.L., Wang L., Liu X.X., Cheng M., Qu Y., Xiao H.B. (2015). Comparative pharmacokinetics of gastrodin in rats after intragastric administration of free gastrodin, parishin and *Gastrodia elata* extract. J. Ethnopharmacol..

[3] Zhao Y., Gong X.J., Zhou X., Kang Z.J. (2014). Relative bioavailability of gastrodin and parishin from extract and powder of Gastrodiae Rhizoma in rat. J. Pharm. Biomed. Anal..

[4] Tang C.L., Wang L., Li J.J., Liu X.X., Cheng M.C., Xiao H.B. (2015). Analysis of the metabolic profile of parishin by ultra-performance liquid chromatography/quadrupole-time of flight mass spectrometry. Biomed. Chromatogr..

[5] Huang N.K., Chern Y., Fang J.M., Lin C.I., Chen W.P., Lin Y.L. (2007). Neuroprotective principles from *Gastrodia elata*. J. Nat. Prod..

[6] Hsieh C.L., Chiang S.Y., Cheng K.S., Lin Y.H., Tang N.Y., Lee C.J., Pon C.Z., Hsieh C.T. (2001). Anticonvulsive and free radical scavenging activities of *Gastrodia elata* Bl. in kainic acid-treated rats. Am. J. Chin. Med..

[7] Jang Y.W., Lee J.Y., Kim C.J. (2010). Anti-asthmatic activity of phenolic compounds from the roots of *Gastrodia elata* Bl. Int. Immunopharmacol..

[8] Su S., Wang T., Duan J.A., Zhou W., Hua Y.Q., Tang Y.P., Yu L., Qian D.W. (2011). Anti-inflammatory and analgesic activity of different extracts of *Commiphora myrrha*. J. Ethnopharmacol..

[9] Hwang S.M., Lee Y.J., Kang D.G., Lee H.S. (2009). Anti-inflammatory effect of *Gastrodia elata* rhizome in human umbilical vein endothelial cells. Am. J. Chin. Med..

[10] Ahn E.K., Jeon H.J., Lim E.J., Jung H.J., Park E.H. (2007). Anti-inflammatory and anti-angiogenic activities of *Gastrodia elata* Blume. J. Ethnopharmacol..

[11] Lee J.Y., Jang Y.W., Kang H.S., Moon H., Sim S.S., Kim C.J. (2006). Anti-inflammatory action of phenolic compounds from *Gastrodia elata* root. Arch. Pharm. Res..

[12] Li Z., Wang Y., Ouyang H., Lu Y., Qiu Y., Feng Y., Jiang H., Zhou X., Yang S. (2015). A novel dereplication strategy for the identification of two new trace compounds in the extract of *Gastrodia elata* using UHPLC/Q-TOF-MS/MS. J. Chromatogr. B.

[13] Chen S., Liu J.Q., Xiao H., Zhang J., Liu A. (2016). Simultaneous Qualitative assessment and quantitative analysis of metabolites (phenolics, nucleosides and amino acids) from the roots of fresh *Gastrodia elata* using UPLC-ESI-Triple quadrupole ion MS and ESI-linear ion trap high-resolution MS. PLoS ONE.

[14] Yu S.S., Zhao J., Zheng W.P., Zhao Y. (2010). Neuroprotective effect of 4-hydroxybenzyl alcohol against transient focal cerebral ischemia via anti-apoptosis in rats. Brain Res..

[15] Dai J.N., Zong Y., Zhong L.M., Li Y.M., Zhang W., Bian L.G., Ai Q.L., Liu Y.D., Sun J., Lu D. (2011). Gastrodin inhibits expression of inducible NO synthase, cyclooxygenase-2 and proinflammatory cytokines in cultured LPS-stimulated microglia via MAPK pathways. PLoS ONE.

[16] Zhu L., Guan H., Cui C., Tian S., Yang D., Wang X., Zhang S., Wang L., Jiang H. (2012). Gastrodin inhibits cell proliferation in vascular smooth muscle cells and attenuates neointima formation in vivo. Int. J. Mol. Med..

[17] Shu C., Chen C., Zhang D.P., Guo H., Zhou H., Zong J., Bian Z., Dong X., Dai J., Zhang Y. (2012). Gastrodin protects against cardiac hypertrophy and fibrosis. Mol. Cell. Biochem..

[18] Wang H., Zhang R., Qiao Y., Xue F., Nie H., Zhang Z., Wang Y., Peng Z., Tan Q. (2014). Gastrodin ameliorates depression-like behaviors and up-regulates proliferation of hippocampal-derived neural stem cells in rats: Involvement of its anti-inflammatory action. Behav. Brain Res..

[19] Zhao S., Li N., Zhen Y., Ge M., Li Y., Yu B., He H., Shao R.G. (2015). Protective effect of gastrodin on bile duct ligation-induced hepatic fibrosis in rats. Food Chem. Toxicol..

[20] Jiang L., Dai J., Huang Z., Du Q., Lin J., Wang Y. (2013). Simultaneous determination of gastrodin and puerarin in rat plasma by HPLC and the application to their interaction on pharmacokinetics. J. Chromatogr. B.

[21] Tang C.L., Wang L., Liu X.X., Cheng M.C., Xiao H.B. (2015). Pharmacokinetic study of *Gastrodia elata* in rats. Anal. Bioanal. Chem..

[22] Zeng X., Zhang S., Zhang L., Zhang K., Zheng X. (2006). A study of the neuroprotective effect of the phenolic glucoside gastrodin during cerebral ischemia in vivo and in vitro. Planta Med..

[23] Wang X.L., Xing G.H., Hong B., Li X.M., Zou Y., Zhang X.J., Dong M.X. (2014). Gastrodin prevents motor deficits and oxidative stress in the MPTP mouse model of Parkinson's disease: Involvement of ERK1/2-Nrf2 signaling pathway. Life Sci..

[24] Wang P.H., Zhao L.X., Wan J.Y., Zhang L., Mao X.N., Long F.Y., Zhang S., Chen C., Du J.R. (2015). Pharmacological characterization of a novel gastrodin derivative as a potential anti-migraine agent. Fitoterapia.

[25] Jiang Z., Zheng X., Gong X., Chao Z., Xin Z., Yang Z., Yan Y. (2016). Relative tissue distribution and excretion studies of gastrodin and parishin from powder and extract of Gastrodiae Rhizoma in rat by UHPLC-ESI-MS/MS. Biomed. Chromatogr..

[26] Liu Z., Wang A.M., Chi M.Y., Wang Y.L. (2012). Quality evaluation of tall *Gastrodia* capsules by simultaneous determination of the contents of multi-constituents. Chin. Pharm. J..

[27] Tang C.L., Wang L., Cheng M.C., Zhang X.Z., Liu X.Y., Xiao H.B. (2014). Rapid and sensitive analysis of parishin and its metabolites in rat plasma using ultra high performance liquid chromatography-fluorescence detection. J. Chromatogr. B.

