# Supplementary material for: An Optimized and Sensitive Pharmacokinetic Quantitative Method of Investigating Gastrodin, Parishin, and Parishin B, C and E in Beagle Dog Plasma using LC-MS/MS after Intragastric Administration of Tall Gastrodia Capsules
Source: Molecules. 2017 Nov 10;22(11):1938. doi: 10.3390/molecules22111938 (PMC6150220; doi:10.3390/molecules22111938)
Supplement: Supplementary file 1 [file molecules-22-01938-s001.pdf]

Table S1 Intensity of gastrodin, parihshin, parishin B, parishin C and parishin E under different experimental condition.

Experimental condition	Gastrodin	Parishin	Parishin B	Parishin C	Parishin E
A-water	85797	52953	0	0	45622
A-0.1% formic acid-water	84372	46455	23657	28816	97947
A-50 mmol/L ammonium formate-watert	65131	38226	0	0	20526
B-2% acetonitrile	1364	2356	280	65	337
B-3% acetonitrile	1170	2514	275	72	353
B-4% acetonitrile	767	2571	276	81	360
B-5% acetonitrile	230	2620	253	74	398
C-0.05% formic acid water	28360	57096	16646	23284	Bifurcated Peak
C-0.1% formic acid water	22189	40507	16576	22671	Bifurcated Peak
C-0.2% formic acid water	28928	50633	13575	18347	98275
C-0.3% formic acid water	19115	26523	5730	7829	38735
C-0.4% formic acid water	24784	43486	10214	14486	77956
C-0.5% formic acid water	22368	37487	8988	11933	64381

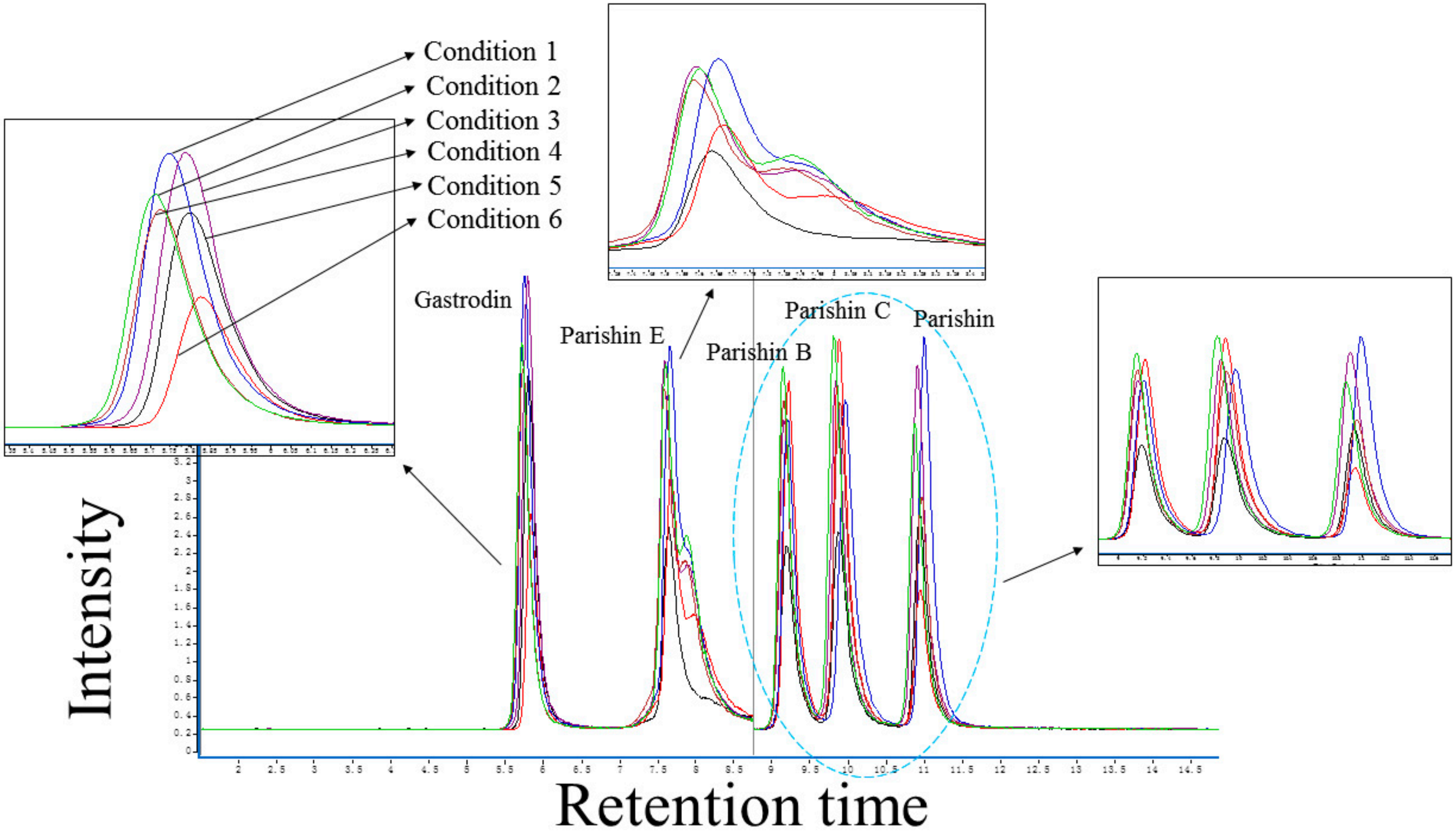

Figure S1 Total ions mass spectrograms obtained from different ion source parameters.
